# A Buried Thermal Rail (BTR) Technology to Improve Electrothermal Characteristics of Complementary Field-Effect Transistor (CFET)

**DOI:** 10.3390/mi14091751

**Published:** 2023-09-07

**Authors:** Zhecheng Pan, Tao Liu, Jingwen Yang, Kun Chen, Saisheng Xu, Chunlei Wu, Min Xu, David Wei Zhang

**Affiliations:** 1 School of Microelectronics, Fudan University, Shanghai 200433, China; 20212020008@fudan.edu.cn (Z.P.); 18112020011@fudan.edu.cn (J.Y.); 18112020016@fudan.edu.cn (K.C.); ssxu@fudan.edu.cn (S.X.); wuchunlei@fudan.edu.cn (C.W.); 2 Shanghai Integrated Circuit Manufacturing Innovation Center Co., Ltd., Shanghai 201203, China

**Keywords:** complementary field-effect transistor (CFET), technology computer-aided design (TCAD), self-heating effect (SHE), power delivery network (PDN), buried power rail (BPR)

## Abstract

The complementary field-effect transistor (CFET) with N-type FET (NFET) stacked on P-type FET (PFET) is a promising device structure based on gate-all-around FET (GAAFET). Because of the high-density stacked structure, the self-heating effect (SHE) becomes more and more severe. Buried thermal rail (BTR) technology on top of the buried power rail (BPR) process is proposed to improve heat dissipation. Through a systematical 3D Technology Computer Aided Design (TCAD) simulation, compared to traditional CFET and CFET with BPR only, the thermal resistance ($R_{th}$) of CFET can be significantly reduced with BTR technology, while the drive capability is also improved. Furthermore, based on the proposed BTR technology, different power delivery structures of top-VDD–top-VSS (TDTS), bottom-VDD–bottom-VSS (BDBS), and bottom-VDD–top-VSS (BDTS) were investigated in terms of electrothermal and parasitic characteristics. The $R_{th}$ of the BTR-BDTS structure is decreased by 5% for NFET and 9% for PFET, and the $I_{on}$ is increased by 2% for NFET and 7% for PFET.

## 1. Introduction

Due to the insurmountable technical challenges of the silicon-based physical layer, as well as the current social development of the era, the demand for computing performance of consumer electronic products tends towards saturation. The semiconductor miniaturization process will be gradually slowed down, which has become a general consensus in the industry. The semiconductor miniaturization process marked by the ‘technology node’ has been extended from the original 18-month cycle to a 24-month cycle [1]. At the same time, the industry is constantly targeting small-size devices in the range of a few nanometers to improve the IC performance, such as Fin field-effect transistor (FinFET) [2,3] and Gate-All-Around Nanosheet FET (GAA-NSFET) [4,5]. Nowadays, using basic rules alone is no longer enough to scale standard cells. It is expected that in the near future, NFET and PFET can be stacked on top of each other for further scaling. The complementary FET (CFET) is one of the most promising devices, which stacks N-type FET (NFET) on P-type FET (PFET) vertically to form an inverter. Such studies have been verified by many institutions, such as IMEC [6], Intel [7] and Applied Materials [8]. It is recognized that GAA-NSFET will replace FinFET below N2, and sheet-based CFET has been proven to have a better performance than fin-based CFET [3].

Recently, several studies have shown the importance of parasitic capacitance in CFET [9,10,11]. Compared with NSFET, CFET shows the possibility of a better performance in frequency and power because of the lower effective capacitance ($C_{eff}$) [9]. In addition, several studies have shown the importance of SHE in multi-gate transistors such as GAA-NSFET. Their tight geometric construction and difficulty in heat conducting will cause device performance degradation and thermal reliability issues [12,13,14]. Recently, some studies have also been carried out on the electrothermal characteristics of CFET. Device design guidelines for a 3 nm node CFET have been investigated from the perspective of electrothermal characteristics [15]. The self-heating effect (SHE) of the CFET has been investigated, and a cross-coupled thermal network model has been proposed [16].

In almost all previous studies, the issue of electrothermal characteristics in CFET has not been studied comprehensively, which should greatly impact the circuit performance. Due to the longer length of the metal via and higher stacking density, it is expected that the heat dissipation will become more severe. Thanks to the backside power delivery network (BPDN) and buried power rail (BPR) technology [17], there are multiple choices and more spaces for depositing the metal via. However, there are no qualitative analyses of the influence of different via strategies on CFET performance.

For the first time, buried thermal rail (BTR) technology is proposed. Different methods of CFET are compared in terms of electrothermal characteristics and parasitic capacitance. A comparison between different PDN methods with a BTR reveals the performance advantage of CFET architecture. Here, the influence of different parameters on the CFET are well studied.

## 2. Modeling Methodology

**Figure 1:**
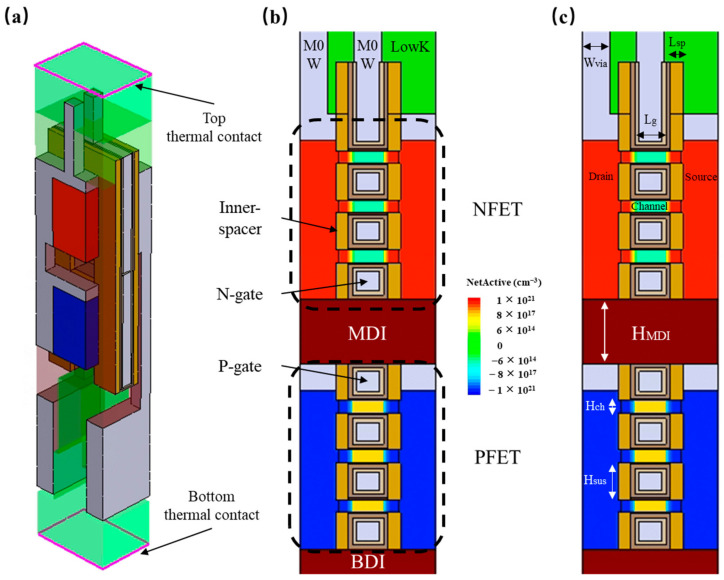
(**a**) Three-dimensional view of the CFET; (**b**) CFET cross-sectional view through the channel; (**c**) schematic of structural parameters of CFET in cross-sectional view.

The CFET was designed based on the studies released from IMEC [18] and the IRDS2022 [1] in the Sentaurus 3-D Technology Computer Aided Design (TCAD) platform. The electrical and structural parameters of CFET were calibrated with the experimental reference of IMEC [19]. Figure 1a shows the 3D view of the CFET. Figure 1b,c show the cross-sectional view of the CFET and its structural parameters. Table 1 shows the electrical and structural parameters of the CFET. The NFET was stacked on the PFET. The gate length was set as 12 nm, while 0.5-nm-thick gate oxide and 1.5-nm-thick ${\mathrm{HfO}}_{2}$ were used. For the gate metal work function, 4.42 eV and 4.84 eV were set to the NFET and PFET, respectively, in order to meet 4 nA/FET off-current. The channel doping concentration of NFET and PFET is $1\times 10^{15}{\;\mathrm{cm}}^{-3}$ and $1\times 10^{18}\;{\mathrm{cm}}^{-3}$, respectively, which is consistent with the calibrated structural parameters and remains constant in subsequent simulations. The source/drain doping concentration of both FETs is $1\times 10^{21\;}{\mathrm{cm}}^{-3}$, whose extension doping concentration is $3\times 10^{20}{\;\mathrm{cm}}^{-3}$. An ohmic contact resistivity between the metal and source/drain was also considered, which was set as $1\times 10^{-9}\;\Omega \cdot {\mathrm{cm}}^{2}$. 

In order to obtain an appropriate heat conduction at the scale of nanodevices, the relevant thermal transmission model in this paper adopts Holland’s model based on the Boltzmann transport equation (BTE), which is calculated using Equation (1) [20]:

(1) $$\kappa =\frac{1}{3}\cdot \underset{j}{\sum }v_{j}{}^{2}\int_{0}^{\frac{\theta_{j}}{T}}C_{V,j}\left(x_{\omega },T\right)\tau_{j}\left(x_{\omega },T\right)dx_{\omega }$$

where $v_{j}$ represents the phonon group velocity, $\theta_{j}$ represents the Debye temperature, $C_{V,j}$ represents the phonon-specific heat per unit volume, and $\tau_{j}$ represents the phonon scattering rate. The subscripts *j* = T, TU, and L represent transverse and longitudinal modes. $x_{\omega }=\omega /k_{B}T$ is the nondimensional phonon frequency, $k_{B}=1.38\times 10^{-23}\;J\cdot K^{-1}$ is the Boltzmann constant, and $\hbar =1.055\times 10^{-34}\;J\cdot s$ is Planck’s constant divided by 2π. Table 2 shows the thermal parameters of the CFET. The top thermal contact resistivity was set as $4\times 10^{-5}{\;\mathrm{cm}}^{2}\cdot K/W$ according to its area to achieve a situation close to the actual environment [21]. Benefitting from the BPDN and BPR technology, the bottom thermal contact was set as the same value as its top, as the backside PDN kept the same metal via density, which should not be a difficult objective to achieve. The thermal resistance between the interfacial oxide and silicon channel was also considered, which was set as $2\times 10^{-4}\;{\mathrm{cm}}^{2}\cdot K/W$ [22]. The global environment temperature was set as 300 K.

In order to simulate the distribution of the lattice temperature, which is caused by the SHE, both the diffusion drift model and thermodynamic model were included. As the thickness of the channel is 5 nm, the quantum confinement effect and the degradation of mobility in the thin layer should not be ignored. Thus, the density gradient quantization model and the thin-layer model were included. The remote Coulomb scattering model, Philips unified mobility model, and enhanced Lombardi model were used to account for the degradation of mobility, which was caused by degraded carrier mobility, electron–hole scattering, and phonon scattering. In a high electric field, the velocity of carriers is confined, so a high-field saturation model was included. The doping-dependent mobility model, bandgap narrowing model, and Shockley–Read–Hall doping dependence model were also adopted.

**Figure 2:**
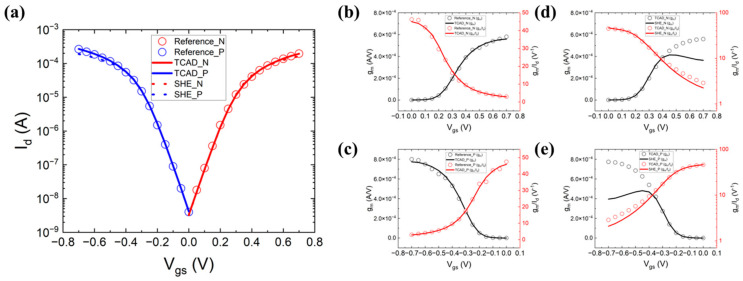
Calibrated curves of double-fin-based CFET between experimental reference and TCAD simulation and curves of double-fin-based CFET with self-heating effect (SHE): (**a**) *I_d_*-*V_gs_*; (**b**) *g_m_*-*V_gs_* and *g_m_*/*I_d_*-*V_gs_* for the NFET; (**c**) *g_m_*-*V_gs_* and *g_m_*/*I_d_*-*V_gs_* for the PFET; (**d**) *g_m_*-*V_gs_* and *g_m_*/*I_d_*-*V_gs_* for the NFET with SHE; (**e**) *g_m_*-*V_gs_* and *g_m_*/*I_d_*-*V_gs_* for the PFET with SHE. (Reference_N means the reference data of the NFET, TCAD_N means the TCAD simulation result of the NFET, SHE_N means the TCAD simulation result of the NFET with self-heating effect, and the same applies to the PFET).

**Figure 3:**
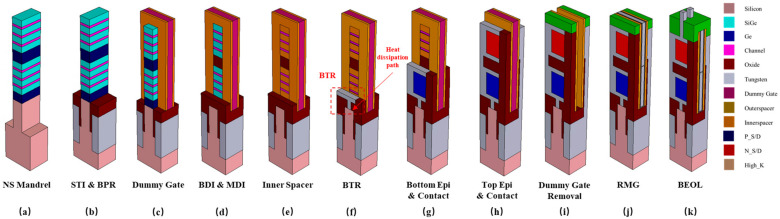
CFET process flow: (**a**) NS Mandrel; (**b**) STI and BPR; (**c**) Dummy Gate; (**d**) BDI (bottom dielectric insulator) and MDI (middle dielectric insulator); (**e**) Inner Spacer; (**f**) BTR; (**g**) Bottom Epi and Contact; (**h**) Top Epi and Contact; (**i**) Dummy Gate Removal; (**j**) RMG (replaced metal gate); (**k**) BEOL (back-end-of-line).

The *I_d_*-*V_g_* curves shown in Figure 2a, the *g_m_*-*V_gs_* and *g_m_*/*I_d_*-*V_gs_* curves for the NFET and PFET shown in Figure 2b,c and the *g_m_*-*V_gs_* and *g_m_*/*I_d_*-*V_gs_* curves for the NFET and PFET with SHE shown in Figure 2d,e ensure the rationality of the device parameter settings of the CFET in a double-fin structure [19]. Reference_N means the reference data of the NFET. TCAD_N means the TCAD simulation result of the NFET. SHE_N means the TCAD simulation result of the NFET with a self-heating effect, and the same applies for the PFET. The work functions of NFET and PFET were adjusted to match the off-current and the threshold voltage. By default, the velocity in the Drift-Diffusion (DD) simulation cannot exceed the saturation value, which is the reason for the underestimation of the drive current. the DD simulations can be adjusted to match the Monte Carlo (MC) simulation results by increasing the saturation velocity in the mobility model. Increasing the $v_{sat}$ value of the NFET and the PFET to $3.21\times 10^{7}\;\mathrm{cm}/s$ and $2.51\times 10^{7}\;\mathrm{cm}/s$, respectively, which are three times the original value, leads to a better fitting of the *I_d_*-*V_g_* curves. The *I_d_*-*V_g_* curves of double-fin-based CFET with SHE are also shown. When the $V_{gs}$ rises, the $I_{d}$ rises. The increment in the $I_{d}$ increases the temperature, which causes the degradation of the $I_{d}$, causing the decrement of the $g_{m}$. The SHE also degrades the device performance, which can be observed by the decrement of the $g_{m}$/$I_{d}$. The calibrated model based on the DD is a simplified scheme to avoid the computationally expensive SHE approach. It is used to provide an approximate solution of the carrier transport, which explains the large differences exhibited in Figure 2d,e. Sheet-based CFET has been proven to have a better performance than fin-based CFET; the following research has been established on sheet-based CFET with similar parameters and models. BTR technology has the potential to improve the performance of the CFET. Figure 3 shows the process flow of sheet-based CFET with BTR.

## 3. Results and Discussion

### 3.1. Buried Thermal Rial

**Figure 4:**
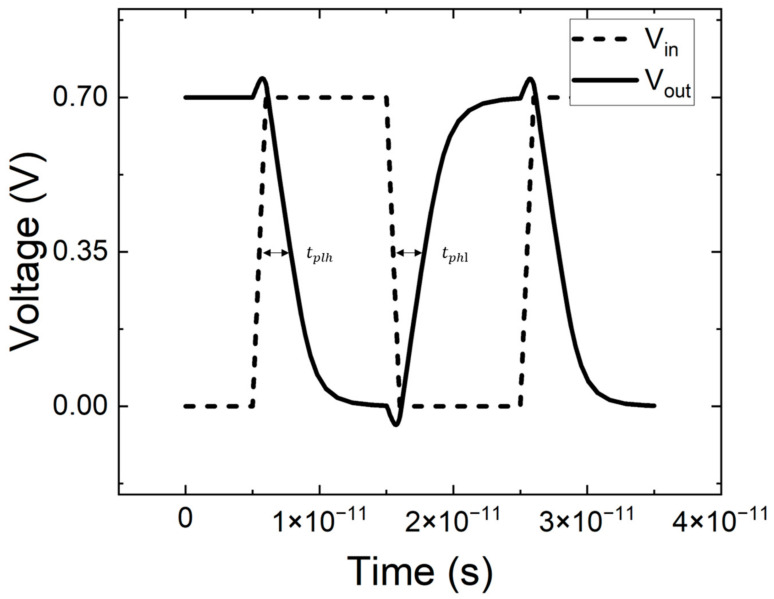
The time–domain dynamic characteristics of the CFET in response to a square-wave input signal.

When treating CFET as an inverter and loading a $1\times 10^{-15}\;F$ output capacitance, the time–domain dynamic characteristics of the CFET in response to a square-wave input signal are shown in Figure 4. It is recognized that the channel stress can be boosted to 4.7 Gpa for NS-PFET to increase the PFET’s on-state current ($I_{on}$) [23]. When the stress of P-channels is 1.4 Gpa, the PFET’s $I_{on}$ attains $2.48\times 10^{-4}\;A$, which is the same as that of the NFET. As shown in Figure 4, the high-to-low delay time ($t_{phl}$) is $2.35\times 10^{-12}\;s$, which is the same as the low-to-high delay time ($t_{plh}$).

The max temperature ($T_{max}$) and thermal resistance ($R_{th}$) are important parameters that reflect the electrothermal characteristics of the CFET. $T_{max}$ is the highest temperature in the CFET, while $R_{th}$ is a parameter that reflects the heat dissipation ability, which is calculated by the expression:

(2) $$R_{th}=\Delta T_{max}/\left(I_{on}\times V_{DD}\right)$$

**Figure 5:**
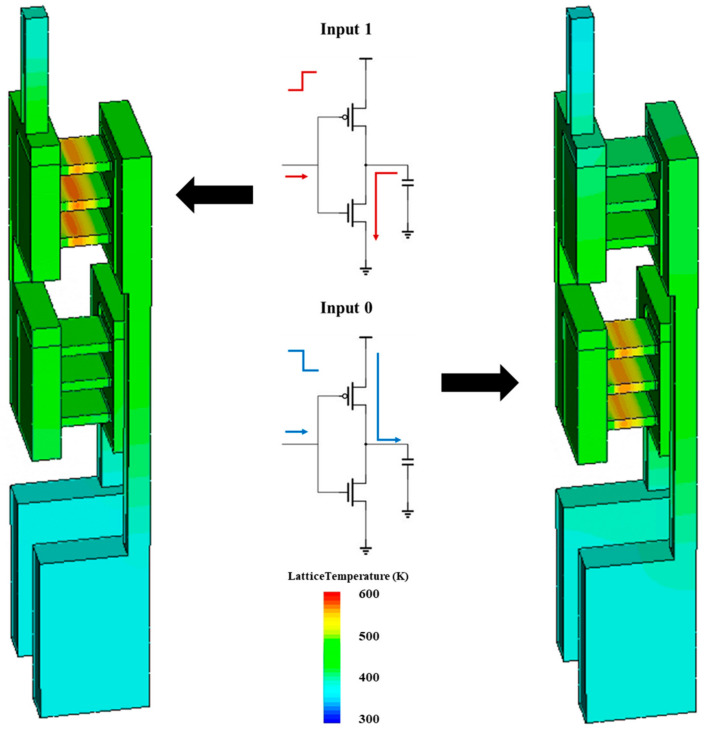
Schematic of the simulation of electrothermal characteristics.

Because the power of the CFET is mainly generated during the period of charging–discharging, it is important to extract the electrothermal characteristics of both NFET and PFET. Figure 5 shows the method for simulating the electrothermal characteristics. When the input is 1, the gate voltage is swept to 0.7 V. The NFET is on, while the PFET is off. Because the capacitance between the out and the ground is pre-charged, the drain of the NFET is 0.7 V. There is a current through the NFET to discharge the capacitance and generate heat in the NFET until the capacitance is fully discharged. When the input is 0, the gate voltage is swept to 0 V. The NFET is off, while the PFET is on. Because the capacitance between the out and the ground is pre-discharged, the drain of the PFET is 0 V. There is a current through the PFET to charge the capacitance and generate heat in the PFET until the capacitance is fully charged.

**Figure 6:**
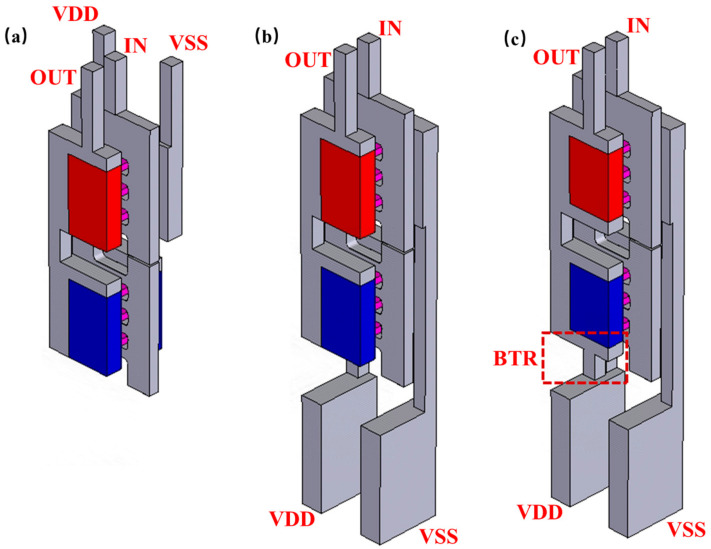
Three CFET structures: (**a**) traditional-CFET, (**b**) BPR-CFET, and (**c**) BTR-CFET.

**Figure 7:**
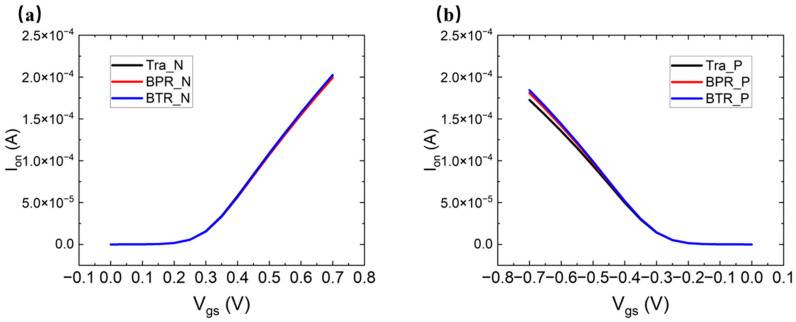
The *I_d_*-*V_g_* curves of these methods: (**a**) NFET and (**b**) PFET.

**Figure 8:**
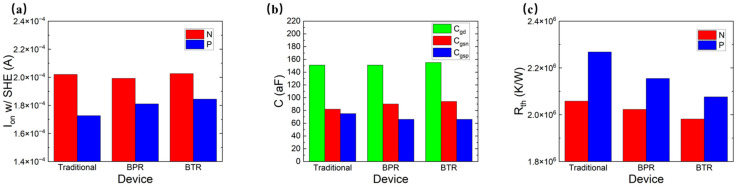
(**a**) The $I_{on}$; (**b**) $C_{gd}$, $C_{gsn}$, and $C_{gsp}$; and (**c**) $R_{th}$ of different methods of the CFET.

Figure 6a–c show three different methods of the CFET, which are the traditional-CFET, the BPR-CFET and the BTR-CFET. Figure 7a,b show the *I_d_*-*V_g_* curves of those methods. As the $V_{gs}$ increases, the degradation to $I_{d}$ occurs due to the higher temperature. As the BPR structure provides a heat dissipation path through the device to the bottom, the $I_{d}$ is improved. As the BTR structure provides a shorter heat dissipation path through the device to the bottom, the $I_{d}$ is better improved. Figure 8a–c show the parasitic capacitance between the gate and the drain ($C_{gd}$), the parasitic capacitance between the gate and the source of the NFET ($C_{gsn}$), the parasitic capacitance between the gate and the source of the PFET ($C_{gsp}$), the $I_{on}$ and the $R_{th}$ of those methods. The $C_{gsp}$ is higher than the $C_{gsn}$ because of the longer distance of the VDD than that of the VSS, which is obvious in the BPR-CFET and BTR-CFET. The BPR creates another low-thermal-resistance path from the middle to the bottom and decreases the total $R_{th}$. Both for the NFET and PFET, the hot spot is much closer to the drain, and the heat flux mainly dissipates through the inner spacer near the drain. For the drain, only one metal via dissipates the heat flux from middle to top, while the thermal resistance between the drain and the bottom is high. Compared with the traditional-CFET, the $R_{th}$ of the BPR-CFET is reduced by 2% for NFET and 5% for PFET, and its $I_{on}$ is decreased by 1% for NFET and increased by 5% for PFET.

We propose a BTR technology that creates another low-thermal-resistance path from the drain side to the bottom, decreasing the thermal resistance between the drain and the bottom. Powered by the BTR technology, the $R_{th}$ of all methods is extremely reduced and the $I_{on}$ is increased. Compared with the traditional-CFET, the $R_{th}$ of the BTR-CFET is reduced by 4% for NFET and 9% for PFET, and its $I_{on}$ is increased by 2% for NFET and 7% for PFET.

### 3.2. Power Delivery Network

**Figure 9:**
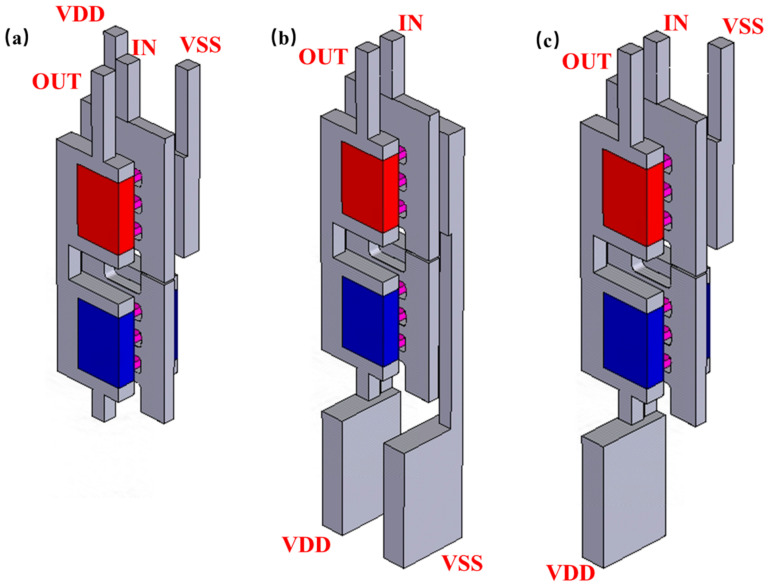
Power delivery structures based on the BTR process: (**a**) BTR-TDTS, (**b**) BTR-BDBS, and (**c**) BTR-BDTS.

**Figure 10:**
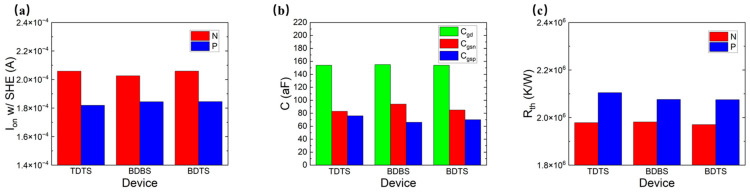
(**a**) The $I_{on}$, (**b**) the $C_{gd}$, the $C_{gsn}$, the $C_{gsp}$ and (**c**) the $R_{th}$ of different methods of PDN.

Figure 9a–c show three different methods of the PDN, which are the BTR-TDTS (top-VDD–top-VSS), BTR-BDBS (bottom-VDD–bottom-VSS) and BTR-BDTS (bottom-VDD–top-VSS) with the BTR. Figure 10a–c show the $C_{gd}$, $C_{gsn}$, $C_{gsp}$, $I_{on}$ and $R_{th}$ of those methods. The BTR-BDTS makes the difference between the $C_{gsn}$ and $C_{gsp}$ smaller than that of the BTR-BDBS, which provides the potential for capacitor matching. Powered by the BTR, all cases are improved on the $I_{on}$ and the $R_{th}$ for the NFET and the PFET. Compared with the BTR-TDTS, the $R_{th}$ of the BTR-BDBS is almost the same for NFET and is decreased by 2% for PFET, and its $I_{on}$ is decreased by 2% for NFET and increased by 2% for PFET.

Because a low-thermal-resistance path from the middle to the bottom is created by the BTR, two low-thermal-resistance paths from the middle to the top created by the VDD and the VSS appear superfluous in the BTR-BDBS. The BDTS creates another low-thermal-resistance path from the middle to the top. Compared with the BTR-TDTS, the $R_{th}$ of the BTR-BDTS is decreased by 1% for NFET and 2% for PFET, and its $I_{on}$ is almost the same for NFET and increased by 2% for PFET. Compared with the traditional-TDTS, the $R_{th}$ of the BTR-BDTS is decreased by 5% for NFET and 9% for PFET, and its $I_{on}$ is increased by 2% for NFET and 7% for PFET.

### 3.3. Characteristics of CFET for Different Dimension Parameters

To further study the electrothermal characteristics, the $C_{gd}$, $C_{gsn}$, $C_{gsp}$, $I_{on}$, $\Delta I_{on}\%$, $R_{th}$ and $\Delta R_{th}\%$ of the CFET with different parameters were extracted. $\Delta I_{on}\%$ is a parameter that reflects the variation in current caused by BTR, which is calculated by the expression:

(3) $$\Delta I_{on}\%=\left|I_{on,\;BTR}-I_{on,\;BPR}\right|\times 100\%$$

$\Delta R_{th}\%$ is a parameter that reflects the variation in thermal resistance caused by BTR, which is calculated by the expression:

(4) $$\Delta R_{th}\%=\left|R_{th,\;BTR}-R_{th,\;BPR}\right|\times 100\%$$

**Figure 11:**
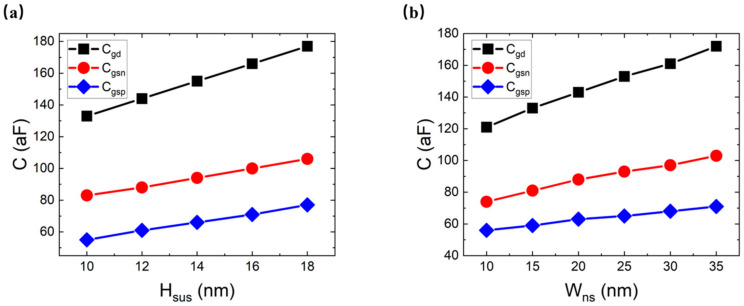
The $C_{gd}$, $C_{gsn}$ and $C_{gsp}$ for different values of (**a**) $H_{sus}$ and (**b**) $W_{ns}$.

Figure 11a,b show the $C_{gd}$, $C_{gsn}$ and $C_{gsp}$ for different values of the channel suspension height ($H_{sus}$) and nanosheet width ($W_{ns}$). The increment in the $H_{sus}$ increases the $C_{gd}$, $C_{gsn}$ and $C_{gsp}$ because the increment in the $H_{sus}$ extends the gate size in the vertical direction. The increment in the $W_{ns}$ increases the $C_{gd}$, $C_{gsn}$ and $C_{gsp}$ because the extension of the gate size in the lateral direction increases the area of the capacitance plate. 

**Figure 12:**
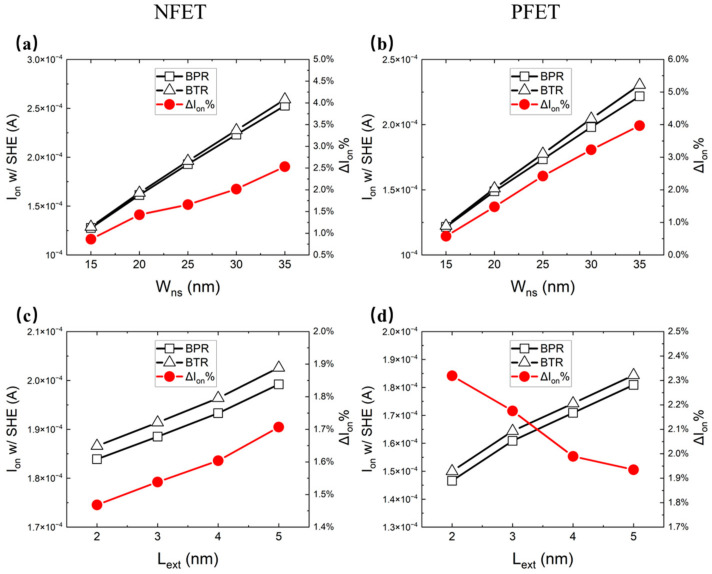
The $I_{on}$ with SHE and the $\Delta I_{on}\%$ of the NFET for different values of (**a**) $W_{ns}$ and (**c**) $L_{ext}$, and those of the PFET for different values of (**b**) $W_{ns}$ and (**d**) $L_{ext}$.

Figure 12a–d show the $I_{on}$ with SHE and the $\Delta I_{on}\%$ for different values of the $W_{ns}$ and extension doping length ($L_{ext}$) between the BTR and BPR. The increment in the $W_{ns}$ and $L_{ext}$ increases the $I_{on}$ due to the extension of the effect channel width and the decrement in the channel resistance. The BTR shows more advantages for the $I_{on}$ than the BPR both for the NFET and PFET. When the $W_{ns}$ increases, the $\Delta I_{on}\%$ increases because of the larger thermal conductivity area. When the $L_{ext}$ increases, the $\Delta I_{on}\%$ of the NFET increases. This is because the $\Delta I_{on}/\Delta T$ is larger at high temperatures. When the $L_{ext}$ increases, the $\Delta I_{on}\%$ of the PFET decreases. This is because a lower $L_{ext}$ contributes more to decreasing the temperature.

**Figure 13:**
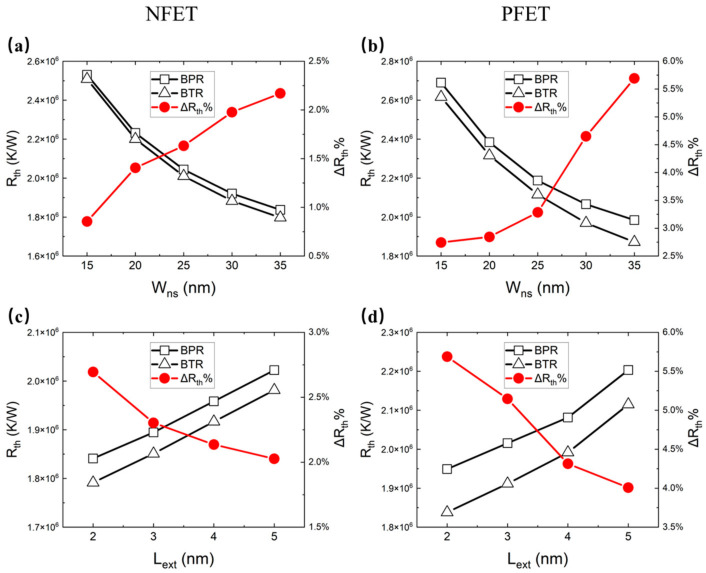
The $R_{th}$ and $\Delta R_{th}\%$ of the NFET for different values of (**a**) $W_{ns}$ and (**c**) $L_{ext}$, and those of the PFET for different values of (**b**) $W_{ns}$ and (**d**) $L_{ext}$.

**Figure 14:**
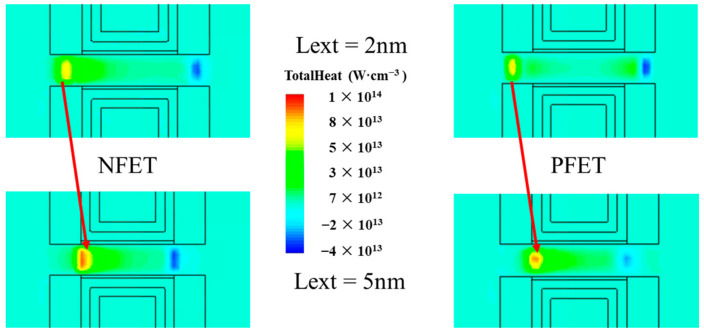
Red arrows show the variation in the hot spot, depending on the $L_{ext}$.

Figure 13a–d show the $R_{th}$ and $\Delta R_{th}\%$ for different values of $W_{ns}$ and $L_{ext}$ between the BTR and BPR. The increment in the $W_{ns}$ lowers the $R_{th}$ because of the extension of the channel’s heat dissipation area. The increment in the $L_{ext}$ strongly increases the $R_{th}$ because of the variation in the hot spot, which increases the heat dissipation path in the high thermal resistance channel, as shown in Figure 14. When the $W_{ns}$ increases, the $\Delta R_{th}\%$ increases because of the larger thermal conductivity area. When the $L_{ext}$ increases, the $\Delta R_{th}\%$ of the NFET decreases. This is because the hot spot is further away from the BTR.

## 4. Conclusions

For the first time, BTR technology is proposed and is shown by a process flow. Different methods of CFET are compared in terms of electrothermal characteristics and parasitic capacitance. Powered by BTR technology, the $R_{th}$ of all methods is extremely reduced, and the $I_{on}$ is increased. Compared with the traditional-CFET, the $R_{th}$ of the BTR-CFET is reduced by 4% for NFET and 9% for PFET, and its $I_{on}$ is increased by 2% for NFET and 7% for PFET. A comparison between different PDN methods with a buried thermal rail (BTR) reveals the performance advantage of the CFET architecture. Compared with the BTR-TDTS, the $R_{th}$ of the BTR-BDTS is decreased by 1% for NFET and 2% for PFET, and its $I_{on}$ is almost the same for NFET and increased by 2% for PFET. Here, the influence of different parameters, such as $C_{gd}$, $C_{gsn}$, $C_{gsp}$, $I_{on}$, $\Delta I_{on}\%$, $R_{th}$ and $\Delta R_{th}\%$, on the CFET is well studied. The increment in $H_{sus}$ and $W_{ns}$ increases the $C_{gd}$, $C_{gsn}$ and $C_{gsp}$. The increment in $W_{ns}$ and $L_{ext}$ increases the $I_{on}$ because of the extension of the effect channel width and the decrement in channel resistance. The increment in the $W_{ns}$ decreases the $R_{th}$ because of the extension of the channel’s heat dissipation area. The increment in the $L_{ext}$ significantly increases the $R_{th}$ because of the variation in the hot spot, which increases the heat dissipation path in the high thermal resistance channel.

## Figures and Tables

**Table 1:** Structural and electrical parameters of the CFET.

Symbol	Quantity	Values
$L_{g}$	Gate length	12 nm
$H_{ch}$	Channel height	5 nm
$H_{sus}$	Suspension height	14 nm
$T_{HfO_{2}}$	Gate ${\mathrm{HfO}}_{2}$ thickness	1.5 nm
$T_{SiO_{2}}$	Gate ${\mathrm{SiO}}_{2}$ thickness	0.5 nm
$L_{sp}$	Spacer length	5 nm
${CD}_{BPR}$	CD of BPR	36 nm
$H_{BPR}$	Height of BPR	90 nm
$D_{BPR}$	Buried depth of BPR	23 nm
$H_{MDI}$	Height of MDI	20 nm
$D_{STI}$	STI depth	70 nm
$M_{0}$	Metal 0 pitch	21 nm
$M_{1}$	Metal 1 pitch	42 nm
$N_{chn}$	N-channel doping concentration	$1\times 10^{15}{\;\mathrm{cm}}^{-3}$
$N_{chp}$	P-channel doping concentration	$1\times 10^{18}\;{\mathrm{cm}}^{-3}$
$N_{sd}$	S/D epitaxy doping concentration	$1\times 10^{21}{\;\mathrm{cm}}^{-3}$
$N_{ext}$	Extension doping concentration	$1\times 10^{20}{\mathrm{cm}}^{-3}$
$R_{sd}$	S/D contact resistivity	$1\times 10^{-9\;}\Omega \cdot {\mathrm{cm}}^{2}$
$SD_{n}$	NFET S/D material	Si
$SD_{p}$	PFET S/D material	SiGe (75% Ge)
$Stress_{p}$	P-channel stress	0.8 Gpa

**Table 2:** Thermal parameters of the CFET.

Thermal Conductivity	$\mathrm{Values}\;\left(W/K\cdot m\right)$
Oxide	1.4
Tungsten	175
Nitride	18.5
${\mathrm{HfO}}_{2}$	2.3
Substrate	148
Channel	7.5
S/D (Si)	5.5
S/D (SiGe)	1.0
**Thermal Contact Resistance**	** $\mathrm{Values}\;\left(cm^{2}\cdot K/W\right)$ **
$\mathrm{Si}/{\mathrm{HfO}}_{2}$	$2\times 10^{-4}$
Top	$4\times 10^{-5}$
Bottom	$4\times 10^{-5}$
Environment Temperature	300 K

## Data Availability

The data that support the findings of this study are available from the corresponding author upon reasonable request.
